# Supplementary material for: Induction of Kanizsa Contours Requires Awareness of the Inducing Context
Source: PLoS One. 2016 Aug 12;11(8):e0161177. doi: 10.1371/journal.pone.0161177 (PMC4982671; doi:10.1371/journal.pone.0161177)
Supplement: S1 Appendix — The priming experiment is an indirect test of unconscious processing of illusory contours. This serves as an illustration that such indirect tests cannot provide conclusive evidence for or against unconscious processing. (DOCX) [file pone.0161177.s001.docx]

**S1 Appendix**

**Introduction**

**Priming experiment**

Previous experiments used illusory contours to capture attention in a visual search task [1] or for priming [2,3]. In our priming experiment, we therefore tested if Kanizsa triangles rendered invisible using a similar masking procedure could be used as primes in a visual search task on a subsequent, visible stimulus array. Like experiment 1, this experiment comprised two consecutive tasks. The first, again called ‘Kanizsa’ task, was an indirect test that investigated whether priming with a Kanizsa triangle in the location of the target improves detection of the target. A second task consisted of a ‘Visibility’ task that directly tested the effectiveness of the masking technique.

**Study design**

In the context of this experiment, priming refers to the attentional cue provided by the prime shape about the location of the subsequently presented target in one of four possible locations that in turn affords a boost in behavioral performance on an orthogonal discrimination task. In the primed conditions the location of the target was always identical with the location of the prime, whereas in the unprimed conditions the prime shape was replaced by a control stimulus, which looked exactly like the distractors in the other three locations. Therefore, the unprimed condition provided a baseline that allowed the assessment of whether the triangle prime boosted the participants’ performance by capturing their attention.

We tested priming with a 2x2x2 design with attentional priming (primed vs. unprimed), the type of priming triangle (real vs. illusory), and prime visibility (invisible vs. visible) as within subject factors. There were two different prime shapes: a real triangle defined by a strong luminance contrast or an illusory (Kanizsa) one. The visibility of the cue was manipulated by varying presentation times of the priming array that followed the mask. A short presentation time of 16.7ms was selected for the invisible conditions to render the prime invisible. In the visible conditions, however, the priming array was presented for 300ms, which left enough time for the prime to enter awareness.

Every participant completed two different tasks. Each task comprised 20 blocks of trials, where one block consisted of 32 trials. Across one task, the unprimed visible and invisible conditions appeared for a total of 160 times each, whereas the four primed conditions (real vs illusory and visible vs invisible) occurred 80 times each. Thus, half the block consisted of unprimed trials and the other half of primed ones.

Behavioral responses were given on a forced-choice discrimination task by button press (left or right arrow) on a standard computer keyboard. Each trial required either a left or a right response (see experimental procedure) and each response type appeared twice per block for each condition, except for the unprimed ones where it appeared four times per block. Conditions were selected pseudo-randomly for every trial but were counterbalanced within each block.

**Participants**

Participants in this experiment were recruited among the UCL student population. Seventeen (13 female, age range: 21-32 years, mean age 24.7±2.5 years) normal, healthy participants took part in the experiment, including the two authors. All other participants were unaware of the experimental hypothesis.

**Stimuli**

In half the trials, participants were cued with one of four possible cues. We created shape stimuli by placing four black discs (inducer elements, diameter=1.96°) in the configuration of a square (width=2.8°). In order to produce the illusory shape of a right isosceles triangle sitting on top of the square, a wedge was removed from each inducer element (Pacman). Wedges started at the center of their respective discs and extended to their edges. One wedge measured 90° and was always positioned either in the bottom left or bottom right inducer. The two wedges that formed the apexes of the triangle measured 45°. Finally, the wedge of the fourth inducer element measured 90°. This Pacman did not form part of the triangle and was positioned opposite the hypotenuse of the right isosceles triangle. It always faced the outside of the disc configuration. When the inducers were oriented with the gaps forming the three corners of a triangle, an illusory light grey Kanizsa triangle was visible on top of the black discs (Fig S1A, i and ii).

Real triangles were identical to the corresponding illusory shapes except that their contour was defined by a luminance contrast with a black line connecting the corners of the triangle (Fig S1A, iii and iv).

The control stimuli were created by altering the orientations of the three Pacmen by a systematic rotation of 180º. This modification broke the link between these inducers and thus no illusory triangle was perceived (Fig S1B). Control stimuli could also be horizontal mirror images of the illustrated examples.

Stimuli in the search array were right isosceles triangles with a contour defined by luminance (Fig S1C, i and ii). Target stimuli were triangles with the right angle pointing down, either to the left or to the right, while in distractors the right angle pointed up (Fig S1C, iii and iv). Participants were asked to detect the target and respond via button press whether its right angle was pointing to the left or to the right.

The mask consisted of a square of black lines connecting four discs, one at each corner. It was wide enough to cover all four Pacmen, as well as the location of the triangle within them (Fig S1D, i). In the ‘Visibility’ task, there was no search array. Instead, the stimulus array following the prime was a square containing a cross that gave no clue about the location and orientation of the target triangle (Fig S1D, ii).

The luminance of the background was 156 cd/m^2^. The luminance of inducer elements, the contour of the real triangle and the black of the mask was 2 cd/m^2^. Finally, the grey of the mask in the first task was 58.2 cd/m^2^.

Note that although there were differences between stimuli, the individual inducers remained the same and there was no change in the local image properties (i.e. square configuration of the four Pacmen and their position among the other 3 configurations). This enabled an assessment of the different effects of priming with the Kanizsa shape, as compared to the corresponding real triangle and ungrouped Pacmen or distractors in the unprimed conditions.


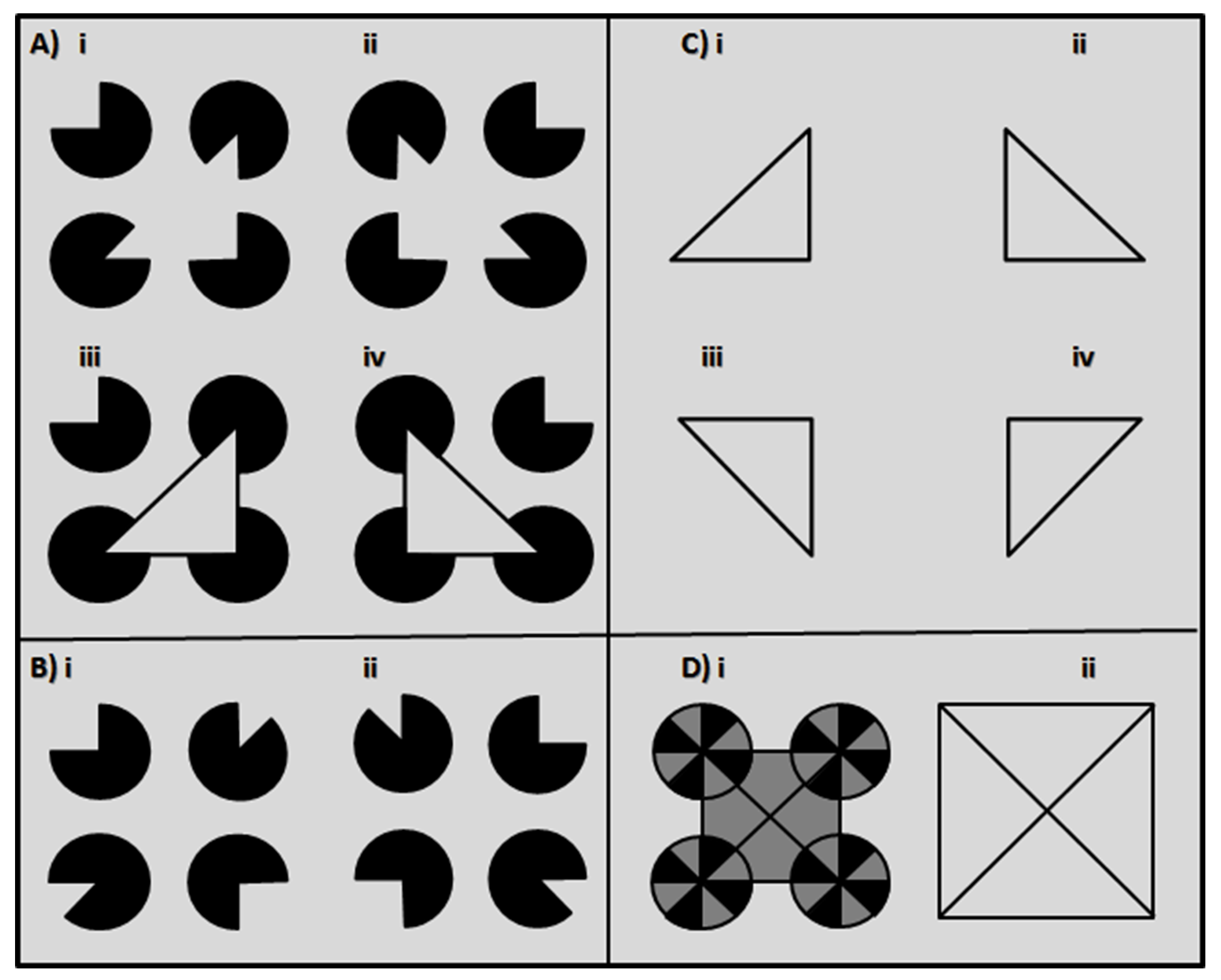


**Fig S1. Illustration of the stimuli in the priming experiment.** A) The four primes were formed of four inducer elements, of which three created either a real triangle or the illusory impression of a triangle. In half the trials, participants were cued with one of the four possible cues: (i) Kanizsa triangle pointing right, (ii) Kanizsa triangle pointing left, (iii) Real triangle pointing right, (iv)Real triangle pointing left. B) Examples of control stimuli used during cue presentation. Control stimuli were created by a systematic 180° rotation of the individual Pacmen, so that all gaps within the black discs pointed to the outside of the formation. Control stimuli could also be the horizontal mirror image of the presented examples. C) The two possible targets in the search task were right isosceles triangles with their right angle pointing down, either to the right (i) or to the left (ii). Distractor stimuli in the search array always pointed up, but varied between pointing left and right. D) (i) The mask used to render the prime invisible. (ii) The uninformative post-stimulus mask replacing the target array in the ‘Visibility’ task.

**Procedure**

**Kanizsa task, indirect test assessing priming**

At first, participants were instructed that they would see an array of four triangles of which only one, the target, had a right angle pointing down and were asked to decide whether this angle was pointing to the left or to the right by pressing the respective response keys.

The 20 experimental blocks were preceded by as many practice blocks as needed in order to fully familiarize the participants with the task requirements. Participants could initiate a new block by pressing any button on the keyboard and, if needed, take small breaks between blocks.

Participants were instructed to fixate a small black dot (0.2° wide) that was present in the center of the screen throughout the experiment. On each trial, the fixation dot was displayed alone for 500ms. Subsequently, a mask array was displayed for 100ms and was immediately followed by one of the priming arrays (see conditions in study design), which was presented for either 16.7ms or 300ms. This array could contain one of the two possible prime shapes (real or illusory triangle) or a simple control stimulus among the other three control stimuli. Immediately after this, a target array containing one target among three distractors was displayed and remained on the screen until the participant’s response.

The response was self-paced; however, participants were instructed to respond as quickly as possible. Responses were made with the index (for left) and the middle or ring finger (for right), depending on how participants felt comfortable. For every response, the fixation dot provided feedback (100ms) by changing its color (green: correct; red: incorrect). Then, the next trial started without any delay. Fig S2A shows the general paradigm for the experimental procedure.

**Visibility task, direct test assessing prime discrimination**

The second task assessed the effectiveness of the mask by measuring the conscious discriminability of the primes in a direct manner. For this purpose, participants were asked to make the same decision as in the previous task, but this time with respect to the prime. Thus, instead of discriminating whether the right angle of the target was pointing to the left or to the right, participants discriminated this aspect of the prime shape.

Procedure, stimuli and timing of the trial sequence in this task were kept identical to the previous indirect test, with a single exception: Instead of a target array, an array comprising new identical mask shapes covered the four stimuli locations (Fig S2B).

**Data analysis**

Response times (in milliseconds) and performance accuracy (in proportion correct) were measured for each of the six conditions. To analyze priming effects, response times were calculated on correct trials only.

For the Kanizsa task, accuracy of responses was less informative, as the target array was present until the participant’s response. We reasoned that priming in the indirect Kanizsa task would be reflected by faster mean response times to primed than unprimed conditions. Therefore the priming effect was calculated by subtracting response times under priming from those without priming for correct responses.


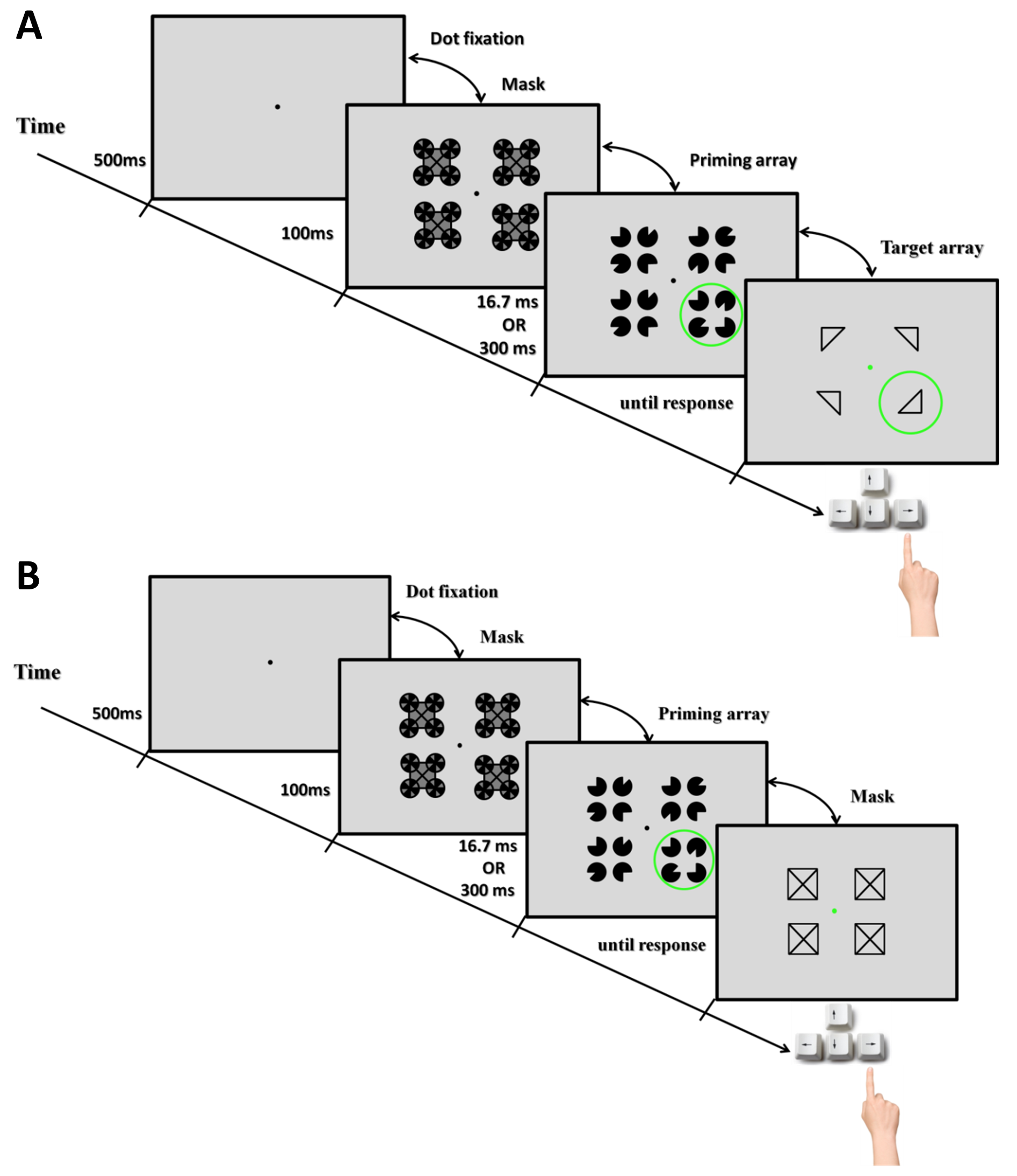


**Fig S2. Illustration of an example trial sequence in the priming experiment.** A) Kanizsa task: Each trial was composed of four frames: fixation period, mask, cueing array and target array. After the participant’s response, the fixation point provided feedback for 100ms (green: correct; red: incorrect). The duration of each frame is shown on the time-line. The duration of the cueing array was varied between conditions: 16.7ms in the invisible conditions and 300ms in the visible ones. Note that in the unprimed conditions, the cueing array did not contain any prime shape and was composed exclusively of control stimuli. B) Visibility task: Trial procedure was identical to the one in the Kanizsa task except for the fourth frame in which the search array was replaced by an array of four identical mask shapes.

Priming effects from all participants for all conditions were entered into a two-way repeated-measures analysis of variance (ANOVA) with prime visibility (visible vs invisible) and prime stimulus (real vs illusory triangle) as within subject factors. In addition to this, further comparisons between experimental conditions (visible or invisible/real or illusory) and control conditions (unprimed) were assessed with paired t-tests. We also determined the significance of the priming effect by comparing it against zero with one-sample t-tests.

In the visibility task, the variable of interest was the accuracy of responses as this allowed us to test whether participants could discriminate the orientation of the primes. One-sample t-tests were carried out at the group level for each condition individually in order to assess whether the participants’ level of performance was significantly above chance (>0.5) and paired-tests were used to compare performance in different experimental conditions. As in experiment 1, for all t-tests we also calculated the default Bayes Factor in addition to frequentist statistics.

**Results**

In the priming experiment we asked whether masked Kanizsa shapes could afford an attentional priming effect. We tested whether a Kanizsa stimulus presented as part of an array of four stimuli could prime participants to the location of a target in a visual search array presented subsequently. The prime could either be a Kanizsa triangle or a triangle defined by a real luminance contour. In the unprimed condition all four stimuli in the priming array were control stimuli without a triangle shape.

**Kanizsa Task**

Overall participants made very few errors in determining whether the target pointed to the left or to the right. Indeed, the mean accuracy in all conditions was above 0.94. Therefore, for each experimental condition we quantified the effect of priming by the difference in response time with priming subtracted from that without priming on correctly answered trials (Fig S3A).


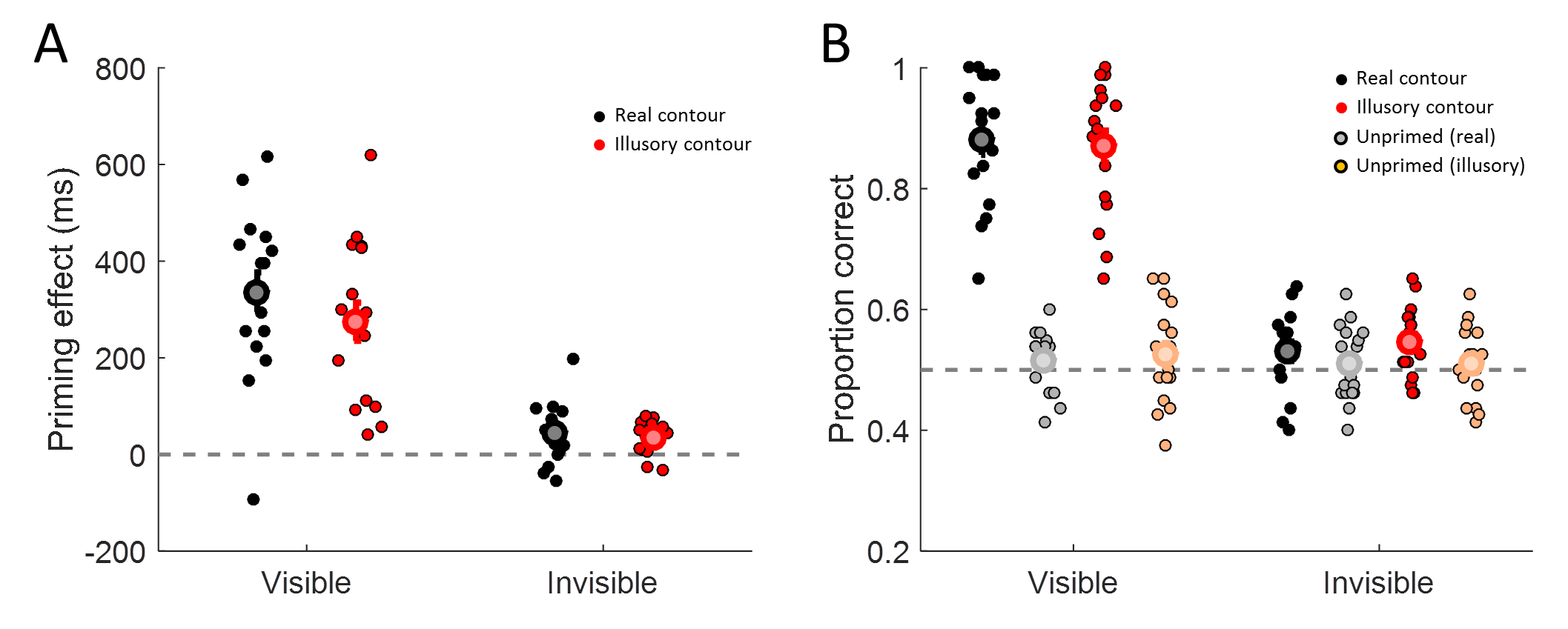


**Fig S3. Results of experiment 2.** Priming effect (response time for unprimed trials minus primed trials) on the Kanizsa task (A) and accuracy for discriminating the inducer location in the Visibility task (B) for visible or invisible (masked) trials. Each dot represents the mean performance of an individual participant in each of the conditions. The large symbols and error bars denote the mean ±1 standard error for each condition. Black: real luminance contour. Red: illusory (Kanizsa) contour. In B) Grey: unprimed trials dummy coded for real contour prime. Orange: unprimed trials dummy coded for illusory contour prime.

We conducted a two-way repeated-measures analysis of variance, with visibility (visible vs invisible) and stimulus (illusory vs real triangle) as within subject factors, to compare the priming effects (response time on unprimed minus primed trials) between conditions. There was a significant main effect of visibility of the prime (*F*(1,16)=60.06, p<0.001), and a significant main effect of stimulus type (*F*(1,16)=13.82, p=0.002). The interaction between visibility and the stimulus type was not significant (*F*(1,16=3.52, p=0.079).

There was a clear difference in the strength of priming between the visible and invisible condition. However, the critical test is whether the priming effect was significant, that is, if it differed from zero (i.e. a paired t-test between primed and unprimed response times). Unsurprisingly, the very pronounced priming effect for visible trials was extremely significant for both conditions (real: *M*=336ms, *t*(16)=8.2, *p*<0.001, *BF_10_*>4.1*10^4^; illusory: *M*=275ms, *t*(16)=6.9, *p*<0.001, *BF_10_*>5.8*10^3^). There was also a much more modest but nonetheless very significant priming effect for both stimulus types in the invisible condition when inducers were masked (real: *M*=43ms, *t*(16)=3, *p*=0.01, *BF_10_*=5.6; illusory: *M*=36ms, *t*(16)=4.4, *p*<0.001, *BF_10_*=80.8).

**Visibility Task**

As in experiment 1 we used a direct Visibility test to assess whether participants might have had residual awareness of the priming stimuli under masking. The task procedure was the same as in the Kanizsa task except that the search array was replaced with a foil in which all four stimuli were identical and non-informative about the correct answer. We quantified the accuracy (proportion correct) with which participants could discriminate the orientation of the priming triangle (Fig S3B).

Unsurprisingly, in the visible condition performance for discriminating the orientation of a real (*M*=0.88, *t*(16)=14.9, *p*<0.001, *BF_10_*=9.5*10^7^) and an illusory triangle (*M*=0.87, *t*(16)=14, *p*<0.001, *BF_10_*=4.0*10^7^) was significantly above chance. Conversely, performance for the unprimed conditions, that is, stimulus arrays that contained no triangle shape, performance was consistently at chance (“real”: *M*=0.52, *t*(16)=1.3, *p*=0.21, *BF_10_*=0.52; “illusory”: *M*=0.53, *t*(16)=1.3, *p*=0.219, *BF_10_*=0.5; but note that these two conditions were dummy coded in this case as they contained the same stimuli).

Critically, performance in the invisible condition showed that discrimination in the primed with an illusory triangle condition was also significantly above chance (*M*= 0.55, *t*(16)=3.24, *p*=0.005, *BF_10_*=9.4), and discrimination of a real triangle showed a similar result even though it did not reach significance (*M*=0.53, *t(*16)=1.9, *p*=0.075, *BF_10_*=1.1). Performance for the two invisible triangles also did not differ significantly (*t(*16)=-0.9, *p*=0.385, *BF_10_*=0.35). Again, as expected both of the unprimed conditions were at chance level (“real”: *M*=0.51, *t(*16)=0.6, *p*=0.534, *BF_10_*=0.3; “illusory”: *M*=0.51, *t(*16)=0.7, *p*=0.494, *BF_10_*=0.31).

**Discussion**

Taken together, the results of the priming experiment contradict those of experiment 1 and 2 , as they suggest that Kanizsa triangles rendered invisible by masking could afford an attentional priming effect in a subsequent visual search task. However, a control experiment testing participants’ ability to discriminate the prime shape orientation directly suggested that this small priming effect for invisible trials could have been due to some residual awareness of the masked triangle stimuli. When conducting such tests of awareness, it is of paramount importance to ensure that trials with masked and unmasked conditions are interspersed. When only masked conditions are tested for awareness, it is possible that the participant’s performance is at chance even though there is in fact residual awareness in the main task due to what has been referred to as “priming of awareness” [4]. Conversely, the ability to correctly identify purportedly subliminal prime stimuli (as in our priming experiment) may have been enhanced by the inclusion of trials with clearly visible primes [5]. Either way, because we controlled for this possibility our visibility test should have provided a robust test of awareness. Therefore, the most parsimonious interpretation of the priming results is that participants had some residual awareness of the primes and that Kanizsa contours were probably not processed when inducers were masked. Using a different masking technique, for example the fast counter-phase flicker used in experiment 2 could be more effective and provide a better test of whether unconscious Kanizsa stimuli can act as attentional cues.

Critically, however, because priming experiments like this are only an indirect test of an effect that is not specific to illusory contour formation, this would not be informative. Even if we interpret the somewhat inconclusive results of this experiment as showing that masked triangles produced priming effects, this does not provide direct evidence that this advantage is actually caused by the presence of illusory contours. A Kanizsa triangle can clearly provide a salient attentional cue but this may be due to shared features, such as the collinearity of the edges in the inducers or the implication of a surface. This is also consistent with previous reports that priming of a shape discrimination task by subliminal stimuli depends on the strength of the salient region [6] and that salient regions of such stimuli are detected efficiently regardless of whether they are bounded by illusory contours [7,8]. Finding a priming effect thus only suggests that attentional cuing can occur without awareness but it does not rule out that other factors produced this attentional capture. Conversely, the absence of a priming effect would only confirm that whatever acts as attentional cue is disrupted. Therefore, the only meaningful test that illusory contours are indeed formed is via a measure that is *specific* to this percept.

**References**

1. Davis G, Driver J. Parallel detection of Kanizsa subjective figures in the human visual system. Nature. 1994;371: 791–793. doi:10.1038/371791a0

2. Barlasov-Ioffe A, Hochstein S. Illusory-contour figures prime matching of real shapes. Perception. 2009;38: 1118–1131.

3. Seydell-Greenwald A, Schmidt T. Rapid activation of motor responses by illusory contours. J Exp Psychol Hum Percept Perform. 2012;38: 1168–1182. doi:10.1037/a0028767

4. Lin Z, Murray SO. Priming of awareness or how not to measure visual awareness. J Vis. 2014;14. doi:10.1167/14.1.27

5. Pratte MS, Rouder JN. A task-difficulty artifact in subliminal priming. Atten Percept Psychophys. 2009;71: 1276–1283. doi:10.3758/APP.71.6.1276

6. Poscoliero T, Marzi CA, Girelli M. Unconscious priming by illusory figures: the role of the salient region. J Vis. 2013;13: 27. doi:10.1167/13.5.27

7. Stanley DA, Rubin N. fMRI activation in response to illusory contours and salient regions in the human lateral occipital complex. Neuron. 2003;37: 323–331.

8. Stanley DA, Rubin N. Rapid detection of salient regions: evidence from apparent motion. J Vis. 2005;5: 690–701. doi:10.1167/5.9.4
